# Detection of County Economic Development Using LJ1-01 Nighttime Light Imagery: A Comparison with NPP-VIIRS Data

**DOI:** 10.3390/s20226633

**Published:** 2020-11-19

**Authors:** Hongliang Liu, Nianxue Luo, Chunchun Hu

**Affiliations:** School of Geodesy and Geomatics, Wuhan University, Wuhan 430079, China; whullliu@whu.edu.cn (H.L.); chchhu@sgg.whu.edu.cn (C.H.)

**Keywords:** LJ1-01, NPP-VIIRS, county-level economic index, random forest (RF) regression

## Abstract

Nighttime light (NTL) remote sensing data have been widely used to derive socioeconomic indicators at the national and regional scales to study regional economic development. However, most previous studies only chose a single measurement indicator (such as GDP) and adopted simple regression methods to investigate the economic development of a certain area based on DMSP-OLS or NPP-VIIRS stable NTL data. The status quo shows the problems of using a single evaluation index—it has a low evaluation precision. The LJ1-01 satellite is the first dedicated NTL remote sensing satellite in the world, launched in July 2018. The data provided by LJ1-01 have a higher spatial resolution and fewer blooming phenomena. In this paper, we compared the accuracy of the LJ1-01 data and NPP-VIIRS data in detecting county-level multidimensional economic development. In three provinces in China, namely, Hubei, Hunan and Jiangxi, 20 socioeconomic parameters were selected from the following five perspectives: economic conditions, people’s livelihood, social development, public resources and natural vulnerability. Then, a County-level Economic Index (CEI) was constructed to evaluate the level of multidimensional economic development, with the spatial pattern of the multidimensional economic development also identified across the study area. The present study adopted the random forest (RF) and linear regression (LR) algorithms to establish the regression model individually, and the results were evaluated by cross-validation. The results show that the RF algorithm greatly improves the accuracy of the model compared with the LR algorithm, and thus is suitable for the study of NTL data. In addition, a better determinate coefficient (R^2^) based on the LJ1-01 data (0.8168) was obtained than that from the NPP-VIIRS data (0.7245) in the RF model, which reflects that the LJ1-01 data offer better potential in the evaluation of socioeconomic parameters and can be used to identify, both accurately and efficiently, multidimensional economic development at the county level.

## 1. Introduction

The availability of artificial lights is often associated with wealth and a modern society due to the increased intensity of urban lights caused by the rapid increase in human activities, traffic construction and urban expansion [1,2]. Previous studies [3,4,5] have shown that, compared with ordinary optical remote sensing images, nighttime light (NTL) data do better to reflect the relationship between human activities and social development and are widely used in socioeconomic parameter prediction, dynamic monitoring of urbanization processes, energy consumption estimation and environmental assessment, etc.

At present, two widely used NTL products are the Defense Meteorological Satellite Program—Operational Linescan System (DMSP-OLS) stable NTL data and Suomi National Polar-orbiting Partnership—Visible Infrared Imaging Radiometer Suite (NPP-VIIRS) NTL data [6]. Obtained by six different satellite sensors, the DMSP-OLS stable annual night light data products include 34 annual images from 1992 to 2013, whereas there are problems such as a lack of radiation calibration, low spatial resolution and signal oversaturation [7]. As a successor to the DMSP-OLS sensor, NPP-VIIRS has offered a series of high-quality imagery of day/night band nighttime lights since 2012 and has provided significant improvements to the DMSP-OLS data, including a wider measurement range, higher spatial resolution and on-board calibration [8,9]. Previous studies have shown that, compared to the DMSP-OLS data, NPP-VIIRS images have a higher accuracy in urban built-up area mapping and socioeconomic parameter estimation [10,11]. LJ1-01, a new generation NTL satellite developed by Wuhan University in China, was successfully launched on 2 June 2018 [12]. LJ1-01 is the first dedicated nighttime light remote sensing satellite in the world and the first low-orbit satellite with earth observation capacity and enhanced satellite navigation [13]. Compared with DMSP-OLS and NPP-VIIRS, LJ1-01 acquires NTL data at a finer spatial resolution (~130 m), with 14-bit quantization and a revisit time of 15 days. The higher spatial resolution of the LJ1-01 data means that more spatial details of the light sources can be displayed [14]. Additionally, the LJ1-01 data do not suffer the same problems of saturation and blooming as the DMSP-OLS data, because the gap in sensor capabilities, lighting sources, are less averaged by the surrounding areas in an LJ1-01 image, which possesses a higher spatial resolution [15]. The specifications of these three types of NTL data are shown in Table 1. These advantages can greatly improve the ability to detect artificial lighting and provide new insights into and possibilities for the studies of human activities, urban construction and social development. Several studies have demonstrated that the LJ1-01 imagery data can provide more capacity in comparison with the NPP-VIIRS NTL data. Jiang et al. [12] confirmed that the LJ1-01 data is more effective in comparison with the NPP-VIIRS data when used for investigating urban light pollution. Li et al. [13] also found that LJ1-01 data can achieve better extraction results for mapping urban areas. Zhang et al. [16] applied the LJ1-01 and NPP-VIIRS NTL data to estimate 10 socioeconomic parameters and concluded that the former can be used as a more effective tool to measure socioeconomic indices. Nevertheless, they only focused on the evaluation of a single-dimensional indicator at the prefecture level and did not conduct a more comprehensive analysis of the county-level economic data. Thus, this research explores the potential of the LJ1-01 NTL data in evaluating comprehensive economic indicators at the county level to better verify the significance and value of the LJ1-01 NTL data in urban development analysis and socioeconomic studies.

Currently, most studies on socioeconomic parameter estimation based on NTL data are conducted at the national-, provincial- or prefecture-level spatial units, while the studies that analyze regional economic development at the county level are relatively rare. As the county is the basic unit of the Chinese administrative division, the county economy is the basic unit of the national economy and occupies an extremely important position in the development of the national economy. In recent years, the county economy has been developing rapidly. Hence, the problem of unbalanced county development has gradually emerged, eventually becoming a hot topic for scholars [17,18,19,20]. Most of the previous studies on regional economic differences mainly selected a single measurement indicator, such as GDP per capita or GDP, and used traditional methods such as standard deviation, coefficient of variation and linear regression to investigate the economic development status in a certain region [21,22]. These studies show that socioeconomic activity is strongly correlated with night light intensity at a large scale (e.g., at the national or provincial level), which can be well described by regression models. However, in recent years, the selection of economic evaluation indicators has changed, gradually transitioning from a single indicator to multidimensional indicators that can do better to reflect the comprehensive economy of a county [23]. The development of the county economy is comprehensively evaluated by combining factor analysis, analytic hierarchy process and principal component analysis, etc. Combined with the exploratory spatial data analysis (ESDA) technology based on GIS, these approaches help people reveal the development of the regional economy and the evolution of the imbalance from a spatial perspective. This makes the difference between the economic models and natural conditions more significant and the relationship between the economic parameters and NTL more complicated at the finer scale (e.g., at the county or town level). As a single global model, classic linear regression may not capture complex relationships correctly [24]. In recent years, machine learning methods have been more commonly used for classification or regression in remote sensing applications due to their high predictive accuracy [25,26,27]. Among various machine learning methods, random forest (RF) is getting prominence for its advantages, such as being able to handle complex relationships, identifying the relationship between dependent variables and independent variables, low sensitivity to noise and being able to avoid overfitting [28,29,30]. The RF regression model has recently been successfully combined with NTL and other multisource data for population gridding and human activity detection [31,32]. However, little research has been applied to the LJ1-01 data; thus, this study aims to explore the application of the RF algorithm to the LJ1-01 and NPP-VIIRS data to detect economic development at the county level. Traditional linear regression (LR) is also compared in this paper.

Our main contributions are summarized as follows: (1) Based on the Entropy-corrected Analytic Hierarchy Process (Entropy-AHP) and the socioeconomic statistical data, the County-level Economic Index (CEI) of each county in the Hubei, Hunan and Jiangxi provinces are calculated to reflect the economic development of the region comprehensively. (2) Several features of nighttime light imagery are selected as multidimensional indicators; then, the random forest algorithm (RF) is used to establish the mathematical model and the results are compared with the results of the traditional linear regression algorithm (LR). Two kinds of NTL (LJ1-01/NPP-VIIRS) data are separately used to regress the CEI to identify the development of the county economy. A comparative analysis of these two result sets is also given. (3) A series of geostatistical methods based on GIS are used to analyze the economic spatial pattern at the county level in the study area, and some reliable and valuable conclusions are drawn. The rest of the paper is organized as follows: Section 2 introduces the materials and methods of this paper, followed by Section 3 and Section 4, which present the analysis and discussion of the results. Finally, Section 5 concludes with the findings of this study and provides prospects for further studies.

## 2. Materials and Methods

### 2.1. Study Area

**Figure 1:**
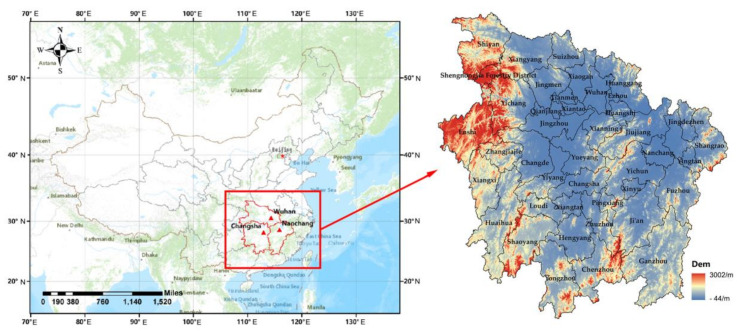
Study area.

As shown in Figure 1, three provinces, Hubei, Hunan and Jiangxi, were selected as the study area for comparison purposes. The study area is located in the central part of the People’s Republic of China, covering longitude 108°21′ E~118°29′ E and latitude 24°29′ N~33°07′ N. The terrain is mainly composed of plains, hills, mountains, rivers and lakes, and the mountains are mainly concentrated in the western and southern regions of the study area and at the junction of Hunan and Jiangxi. By the end of 2019, there were 325 county-level administrative divisions in the study area. The urban agglomeration in the middle Yangtze River, which is composed of the capital cities of these three provinces (Hubei—Wuhan, Hunan—Changsha and Jiangxi—Nanchang, respectively), is an important part of China’s Yangtze River Economic Belt. In 2015, the State Council of China officially approved the “Development Plan for the Urban Agglomeration in the Middle Yangtze River” and identified this area as the key provinces for economic development in the central part of China. However, due to the different location of each county in the study area, the economic development status among these counties has a large spatial difference. There is a large disparity in the county economy, and the development of the county economy is extremely unbalanced. Therefore, for this study we selected these three provinces as the study area to explore the differences in the economic development statuses of these counties.

### 2.2. Data and Data Preprocessing

In this research, a total of 48 phases of NPP-VIIRS monthly composite data from 2015 to 2018 and 2 phases of annual NTL data from 2015 to 2016 provided by the Earth Observation Group, Payne Institute for Public Policy (https://eogdata.mines.edu/download_dnb_composites.html), were selected to calibrate and synthesize the 2018 NPP-VIIRS annual stable NTL data of the study area. The Chinese 2018 synthetic LJ1-01 NTL data were provided by the High-Resolution Earth Observation System of the Hubei Data and Application Center (http://www.hbeos.org.cn/). The data were synthesized using 275 cloudless LJ1-01 images in China from June to December 2018. The nighttime light image matching algorithm was utilized to obtain the tie points, which were used in the planar block adjustment with the ground control points. Then, orthorectification of all images was implemented. Finally, the nighttime light map of China was produced by mosaicking all the nighttime light orthoimages [33]. Therefore, the positioning accuracy of the 2018 synthetic LJ1-01 NTL data has been improved. The socioeconomic statistical data used in this paper are from the Statistical Yearbook of 2019 provided by the provincial statistical bureaus and the 2018 National Economic And Social Development Statistical Bulletins published by each county (http://tjj.hubei.gov.cn, http://tjj.hunan.gov.cn/, http://tjj.jiangxi.gov.cn/). Few instances of missing data were encountered, and we used the average value or the data of the adjacent years to replace a missing value. In addition, some basic geographic information data of China were applied in this research, such as the administrative boundary, DEM (Digital Elevation Model), farmland production potential data, annual average precipitation data and annual average temperature data. The administrative boundary, DEM and farmland production potential data sets came from the Resource and Environment Data Cloud Platform of the Chinese Academy of Sciences (http://www.resdc.cn/Default.aspx). The DEM was generated based on the latest SRTM (Shuttle Radar Topography Mission) V4.1 data after collating and splicing, and its spatial resolution was 90 m. Based on the cultivated land distribution, soil and DEM data, the farmland production potential data for China were made by using the GAEZ (global agroecological zones) model, comprehensively considering various factors such as the light, temperature, water, CO_2_ concentration, agricultural climate constraints and terrain. The annual average precipitation and annual average temperature data in 2018 were synthesized according to the monthly average data, which were obtained from the National Earth System Science Data Center, National Science and Technology Infrastructure of China (http://www.geodata.cn), with a spatial resolution of approximately 1 km. These two sets of monthly data were generated based on the global 0.5° climate data set released by the CRU (Climatic Research Unit) and the high-resolution climate data set released by WorldClim. The data applied in this paper and the related instructions are shown in the Appendix A, Table A2.

#### 2.2.1. NPP-VIIRS Data

The original NPP-VIIRS data include not only the light intensity of the urban area but also some background noise, such as fire, burning exhaust gases and volcanic eruptions [34]. Judging from the raw data, due to the influence of the background noise, some pixels in the NPP-VIIRS data have negative values. In addition, highly reflective surfaces, such as snow-capped mountains, result in sudden increases in pixel values in areas where light is weak [35]. Due to these issues, the original NPP-VIIRS data must be processed before being used to evaluate the CEI. The method proposed by Hu and Zhou to synthesize the NPP-VIIRS NTL data for the China region was performed in this paper. The data processing flow is as follows [36,37]:

Step 1. Annual average image synthesis and negative value elimination. According to Formula (1), the monthly light data from 2015 to 2018 is used to synthesize the annual light data, where *DN*_i_ is the radiation value of the monthly composite product, *N* represents the number of months and *DN*_j_ represents the average light value of a year. Theoretically, the light radiation value should not be less than 0; thus, the negative values in the 2015 synthetic data are replaced according to the 2015 standard annual data. Then, the negative values in the synthetic data of 2016–2018 were replaced by the data of the previous year.

(1) $$DN_{j}=\frac{\sum_{i=1}^{N}DN_{i}}{N}$$

Step 2. Elimination of unstable light sources and background noise. According to the standard annual NTL data of 2015 and 2016, which have removed the background noise, the data of these two phases is converted from floating to integer, the part of *DN* > 0 is set as 1, and the part of *DN* ≤ 0 is set as 0, so as to obtain the binary image. Multiply these two binary images to obtain a synthesized binary image. The part with *DN* = 1 is identified as the area without background noise; that is, the stable light sources area in 2015 and 2016. Then use the synthetic binary image as the calibration standard, using Formula (2) to extract the stable light source area from 2015 to 2018. *DN* represents the stable light source in the annual synthesized data after correction; *DN*_k_ represents the stable light source of the image in a certain year to be corrected; and *DN*_k−1_ represents the stable light source of the binary image one year before the correction year.

(2) $$DN=\left\{\begin{array}{l} 0,DN_{k-1}=0|DN_{k}=0;\\ 1,DN_{k-1}=1\&DN_{k}=1; \end{array}\right.,k>2015$$

Step 3. High value elimination and continuous correction. By extracting the brightness value from the synthesized annual nighttime light data, the maximum brightness threshold for each year from 2015 to 2018 can be obtained after mathematical statistical analysis, which can replace the high value. According to the principle that the light brightness value of the next year is not lower than that of the previous year, based on the synthetic nighttime light data in 2015, the data of the other years are corrected year-by-year according to Formula (3), so as to obtain reliable annual nighttime light data of 2018, where *DN* represents the corrected annual light brightness value; *DN*_x_ represents the annual light brightness value to be corrected; and *DN_x_*_−*1*_ represents the corrected annual light brightness value of the previous year.

(3) $$DN=\left\{\begin{array}{l} {\mathrm{DN}}_{x-1},{\;\mathrm{DN}}_{x}\leq {\mathrm{DN}}_{x-1}\\ DN_{x},{\mathrm{DN}}_{x}>{\mathrm{DN}}_{x-1} \end{array}\right.\begin{array}{cc} & x>2015 \end{array}$$

**Figure 2:**
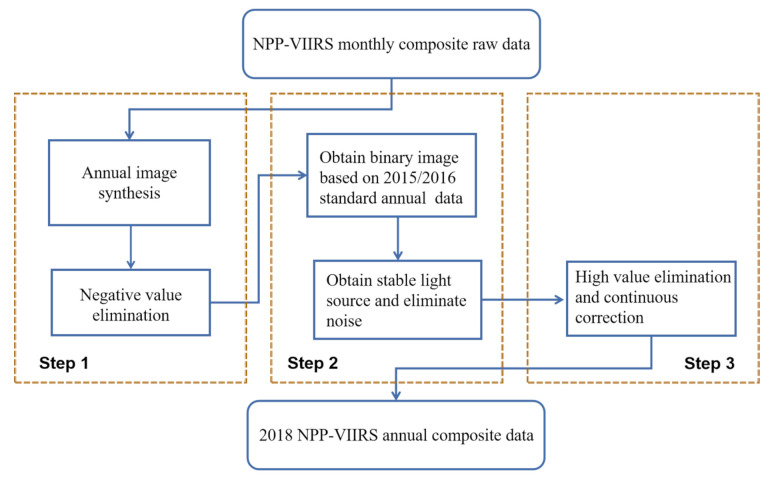
The NPP-VIIRS NTL data processing flow.

**Figure 3:**
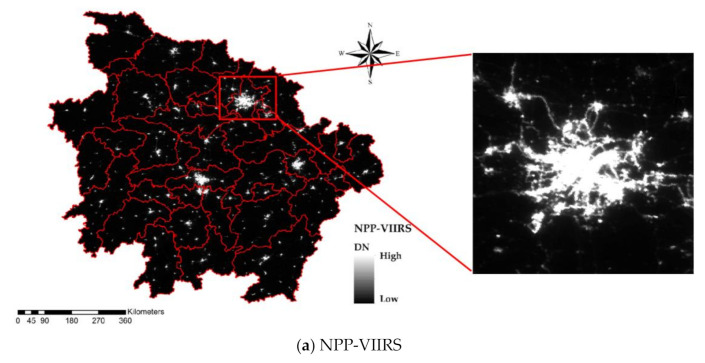

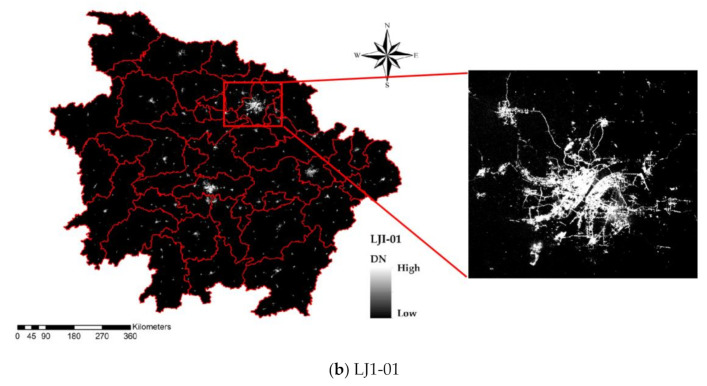
Processed NTL data of the study area. The red rectangle outlines the Wuhan urban area. (**a**) Processed NPP/VIIRS NTL data in 2018 and (**b**) processed LJ1-01 NTL data in 2018.

The NPP-VIIRS data processing flow is shown in Figure 2. The processed 2018 NPP-VIIRS annual composite data in the study area are shown in Figure 3a.

#### 2.2.2. LJ1-01 Data

Nightsat is a concept for a satellite system that enables global observations of the location, extent and brightness of night-time lights at a spatial resolution suitable for the delineation of primary features within human settlements. According to its requirements, lowlight imaging data must achieve minimal detectable radiances with a signal/noise ratio (SNR) of 10 dB [38,39]. The SNR of the LJ1-01 data is higher than 35 dB; consequently, there is no need to remove the noise from the LJ1-01 NTL data, which has no negative values. Nevertheless, it should be noted that the NPP-VIIRS data are calibrated to absolute radiances and provide radiation brightness values. Thus, in order to ensure that the NPP-VIIRS and LJ1-01 NTL can be compared, absolute radiation correction is necessary for the LJ1-01 data. In this paper, the digital numbers (*DN*s) of the LJ1-01 data were converted into radiance to accurately analyze the lighting brightness and discrepancy. The radiation conversion formula is shown as (4), based on lab calibration [15]. The processed 2018 synthetic LJ1-01 NTL data in the study area are shown in Figure 3b.

(4) $$L=DN^{3/2}\cdot 10^{-10},$$

where *DN* is the image gray value of each pixel and L is the absolute radiation corrected value in units of W · m^−2^ sr^−1^ μm^−1^. The radiance of LJ1-01 is converted to the central wavelength, while the NPP satellite uses full-band radiance. Therefore, the difference in bandwidth between the two types of data results in different units of radiation value. For the convenience of comparison, we multiplied the L value by the bandwidth to convert the unit. The radiometric range the LJ1-01 is 460–980 nm, so that the bandwidth is equal to 0.52 μm. After the radiance of each pixel is calculated, the unit is converted to nW · cm^−2^ sr^−1^.

#### 2.2.3. Standardization of Socioeconomic Parameters

In this study, multiple socioeconomic parameters were selected to determine the economic development status of each county. To ensure the comparability among various dimensions, it is necessary to eliminate the measurement differences between various parameters. Therefore, the selected parameters are standardized and normalized according to Formula (5). Based on the fact that the selected indicator will increase or decrease the CEI, it is assigned a respective negative or positive attribute.

(5) $$x_{i}=\left\{\begin{array}{l} \frac{X_{i}-X_{i\min}}{X_{i\max}-X_{i\min}},(X_{i},\mathrm{Positive})\\ \frac{X_{i\max}-X_{i}}{X_{i\max}-X_{i\min}},(X_{i},Negative) \end{array}\right.$$

In the above formula, x_i_ are the dimensionless index values after processing, which are mapped between 0 and 1. *X_i_* represents the original data; *X_i_*_min_ represents the minimum value of index *i*; and *X_imax_* represents the maximum value of index *i.* When the *X_i_* index attribute is positive, the upper formula in (5) is adopted; when the *X_i_* index attribute is negative, the below formula in (5) is adopted.

Multisource geographic basic data with different projection coordinate systems and spatial resolutions are also applied in this study. To reduce these differences, we reproject and resample these data. By using ArcGIS 10.4, all basic geographic data are projected to the Albers equal-area projection coordinate system, which is more suitable for China, and resampled to a spatial resolution of 1000 m, to ensure spatial consistency. Considering the difference in spatial resolution between NPP-VIIRS/LJ1-01, this study only performs the Alberts equal-area coordinate projection processing on them.

### 2.3. Calculation of the County-Level Economic Index

The county economy is the basic unit for evaluating the socioeconomic development of urban and rural areas. In view of the diversity and complexity of the factors affecting the county economy, a series of important indicators affecting the county economy can be selected to evaluate the developmental level of the county economy comprehensively. Due to different perspectives considered by various scholars in the establishment of the county economic evaluation system, there are certain differences in the hierarchical structure design of the system and the selection of the indicators, but the core ideas are basically the same. Generally, the selection of indicators follows the four principles of comprehensiveness, representativeness, accessibility and scientific validity, to ensure the authenticity and objectivity of the evaluation results. Based on the data available in the Statistical Yearbook and the economic indicator system established by previous research [40,41], this research reflects the economic development of each county in the study area from five aspects, including economic conditions, people’s livelihood, social development, public resources, and natural vulnerability, with a total of 20 indicators. The specific evaluation indicators are shown in Table 2.

The weight of the indicators plays an important role in the evaluation of the multidimensional economic development of a county. Therefore, the process of determining the index weight is very important. According to the above indicators, the Analytic Hierarchy Process with Entropy Correction (Entropy-AHP) was used to calculate the CEI of each county. Entropy-AHP is a comprehensive weighted evaluation method. The Analytic Hierarchy Process (AHP) is a multi-index comprehensive evaluation method considering qualitative and quantitative analyses, which can determine the weight according to the intention of the decision makers; while the Entropy Weight Method (EWM), based on the definition of information entropy, objectively calculates the weight of each index and provides the basis for the comprehensive evaluation of multiple indicators without human interference. In view of the advantages and disadvantages of these two methods, the combination of the two methods can make up for the shortcomings of the single method. The specific implementation steps are shown in Formulas (6)–(18).

(1) Calculation of the AHP weight. The AHP decomposes the problem into different factors and combines them into different levels according to their mutual influence to form a multilevel analysis structure model; the relative importance of the lowest level relative to the highest level is determined according to the model. The main steps of the AHP are as follows:
  (a) Construction of the judgment matrix A of the AHP.
    (6) $$A=(a_{ij}{)}_{n\times n}$$
    The element a*_ij_* of the judgment matrix A represents the importance of the *i* index relative to the *j* index, which is given by Santy’s 1–9 scale method [42], in which 1 indicates that the two indices are equally important, 3 indicates that the *i*–th index is more important than the *j*-th index and 9 indicates that the *i*-th index is significantly more important than the *j*-th index. The larger the number, the more important the *i*-th index is relative to the *j*-th index. By using the same scale to subjectively compare the indices with each other, the difficulty of the scoring caused by the different properties or other factors can be reduced to a larger extent, so as to improve the accuracy of the judgment.
  (b) Single-layer weight determination. The normalized eigenvector of matrix A is used as the weight vector *W*. *w_i_* is normalized to obtain the single-level weight of the indicator.
    (7) $$S_{ij}=a_{ij}/\sum_{k=1}^{n}a_{kj},(a_{ij},a_{kj}\in A)$$
    (8) $$\omega_{i}^{}=\sum_{j=1}^{n}S_{ij}$$
    (9) $${\omega_{i}}^{\prime }=\omega_{i}/\sum_{i=1}^{n}\omega_{i}$$
  (c) Consistency verification. Since the construction of the judgment matrix comes from subjective scoring, in order to avoid logical contradictions, it is necessary to verify the consistency of the judgment matrix. The AHP uses the *CI* as the consistency verification index and the *CR* as the consistency ratio. The calculation method is shown in Formulas (10) and (11), where λ is the largest eigenvalue of the matrix and *RI* represents the randomness index. The relationship between the RI and the order of judgment matrix is shown in Table 3. Generally, when the consistency ratio *CR* < 0.1, it is considered that the inconsistency of A is within the allowable range and that the consistency is verified.
    (10) $$CI=\frac{\lambda -n}{n-1}$$
    (11) $$CR=\frac{CI}{RI}$$
  (d) AHP weight determination. The final weight of each index can be calculated by multiplying the second-level index weight by the corresponding first-level index weight obtained in Step (b).
    (12) $$\omega_{AHP-i}=\omega_{i}{}^{\left(1\right)}\cdot \omega_{i}{}^{\left(2\right)}$$
(2) Entropy weight calculation. The entropy is used to calculate the coefficient of variation for each index, which determines the indicator weight and obtains an objective comprehensive evaluation result. The main steps of the entropy-weight method are as follows:
  (e) Indicator assimilation.
    (13) $$P_{ij}=X_{ij}/\sum_{i=1}^{m}X_{ij}$$
    where P_ij_ is the dimensionless value of the *i*-th indicator in the *j*-th county data.
  (f) Calculation of the entropy *e_i_* of each indicator.
    (14) $$e_{i}=-\frac{1}{\ln(m)}\sum_{i=1}^{m}P_{ij}\ln P_{ij}$$
  (g) Calculation of the coefficient of variation of each indicator *g*_i_.
    (15) $$g_{i}=1-e_{i}$$
  (h) Calculation of the weight of each indicator.
    (16) $$\omega_{\mathrm{entropy}}=g_{i}/\sum_{i=1}^{n}g_{i}$$
(3) Calculation of the Entropy-AHP weight. Using *W*_entropy_ to modify *W*_AHP_, the result is normalized to obtain the final *W*_i_.
  (17) $$\beta_{i}=\omega_{\mathrm{AHP}}\times \omega_{\mathrm{entropy}}$$
  (18) $$W_{i}=\beta_{i}/\sum_{i=1}^{n}\beta_{i}$$
(4) Construction of the CEI of the study area. According to the Entropy-AHP weights and the dimensionless data in Section 2.2, linear weighting is used to sum these data. The CEI of each county in the study area is finally obtained according to Formula (19).
  (19) $$CEI=\sum_{i}^{n}W_{i}X_{i}$$

### 2.4. RF Regression Models

Compared with traditional linear regression mathematical models, machine learning algorithms do better to solve nonlinear problems and process high-dimensional data sets. This research provides an example of using the RF algorithm to regress the CEI based on the multidimensional features of the nighttime light images to improve the accuracy of the model. According to the processed NTL data obtained in Section 2.2 and referring to [43], nine feature indicators were selected as the independent variables of the data set from three aspects: the central tendency, dispersion degree and spatial characteristics of the light pixel. The feature indicators of the NTL data are shown in Table 4.

Random forest (RF) is an ensemble learning method that improves the regression tree method by combining a large set of decision trees [29]. Its classifier is CART (Classification And Regression Tree). When the dependent variable of the data set is continuous, the tree algorithm is a regression tree, and the mean value observed by the leaf nodes can be used as the predicted value to solve the regression problem. When the dependent variable of the data set is dispersed, the tree algorithm is a classification tree, which can be used to solve classification problems. The main idea of RF is to use a bootstrap sampling method to extract K sample sets from the original sample N, conduct decision tree modeling for each sample and then predict the data based on the generated multiple trees. The final prediction is determined as the average of the predictions of all the decision-making trees. The classification result is based on the most voted as the final class label; the regression results are determined by the simple average of the results obtained by each tree or the weighted average according to the predicted results of each tree from the training set.

Compared with traditional regression algorithms, RF shows a higher prediction accuracy, as well as the ability to model complex interactions between the different variables without requiring the assumption of a prior probability distribution, and the capacity to analyze the importance of the variables [44]. The main parameters in RF regression modeling include the number of estimators, max features, max depth, min split samples, min leaf samples, etc. More details about the formulated RF regression model can be found in Breiman [29]. As a supervised algorithm, the RF regression model needs to select samples for model training. In this study, the cross-validation method was used to adjust the model error and determine the optimal parameter setting during the modeling. The mean absolute error (MAE) and determinate coefficient (*R^2^*) were used to indicate the accuracy of the model. The formula is as follows:

(20) $$R^{2}=\frac{\sum_{i=1}^{n}{\left(CEI_{e,i}-\bar{CEI}\right)}^{2}}{\sum_{i=1}^{n}{\left(CEI_{t,i}-\bar{CEI}\right)}^{2}}$$

(21) $$MAE=\frac{1}{n}\sum_{i=1}^{n}\left|CEI_{t,i}-CEI_{e,i}\right|$$

where *n* is the number of counties; *CEI_t,i_* is the actual value of county *I*; *CEI_e,i_* is the estimated value of county *I*; and $\bar{CEI}$ is the actual average *CEI* of the counties.

To study the importance of each variable in the CEI calculation model, variable importance analysis was carried out. The importance of the variables is derived from the loss of model prediction accuracy when a variable is randomly permuted [29]. The importance of each variable is measured by calculating the percentage of increase in the root mean square error as the variable permutations change. A higher percentage suggests that the greater error is caused by the random permutation of the variables; thus, the variable is more important.

### 2.5. Spatial Analysis of the CEI

“Everything is related to everything else, but near things are more related than distant things” [45]. Spatial dependency is one of the essential sources contributing to order, pattern and diversity in nature [46]. There are many methods for quantitatively measuring the spatial association features of geographical objects. Global spatial association indices include Moran’s I, Geary’s C, Getis’s G and join count [47,48]. Local spatial association indices include the local Moran’s I, LISA (local indicators of spatial association) and Getis’s G* [49]. In this research, Moran’s I and LISA were selected to quantitatively analyze the spatial pattern of the CEI in the study area, which are effective and feasible.

(1) Moran’s I. Moran’s I is used to measure global spatial autocorrelation. It is a weighted correlation coefficient used to detect departures from spatial randomness, which can indicate spatial patterns. The calculation method is shown in Formula (22) [48], where x_i_ and x_j_ are the values at spatial point i and j, respectively; $\bar{x}$ is the average value of all the points in the entire region; w_ij_ is the weight of the spatial neighborhood relationship; and n is the total number of all spatial statistical units in the study area. The range of Moran’s I is [–1,1]. A positive value means that the object is positively correlated in space, while a negative value means that it is negatively correlated. The statistical significance of Moran’s I can be evaluated by the Z-score after standard normalization.

(22) $$I\;=\frac{n\sum_{i=1}^{n}\sum_{j=1}^{n}w_{ij}{(x}_{i}{-\bar{x})(x}_{j}-\;\bar{x})}{\sum_{i=1}^{n}\sum_{j=1}^{n}w_{ij}\cdot \sum_{i=1}^{n}{{(x}_{i}^{}-\;\bar{x})}^{2}}$$

(2) LISA. Moran’s I can only measure the spatial correlation of the entire region; it cannot reflect the spatial clustering of each partition in the case of spatial heterogeneity. In 1995, American regional economist Anselin proposed the LISA (local indicators of spatial association) statistic to evaluate the existence of clusters in the spatial arrangement of a given variable [49]. This statistic detects local spatial association and can be used to identify local clusters (i.e., regions where adjacent areas have similar values) or spatial outliers (i.e., areas distinct from their neighbors). The calculation of the LISA statistics is shown in Formula (23), where x_i_, x_j_, and w_ij_ have the same meaning as in (22). LISA statistics decompose Moran’s I into contributions for each location. The sum of I_i_ for all observations is proportional to Moran’s I.

(23) $$I_{i}=\frac{{n(x}_{i}-\;\bar{x})\sum_{j=1}^{n}w_{ij}{(x}_{j}-\;\bar{x})}{\sum_{j=1}^{n}{{(x}_{j}-\;\bar{x})}^{2}}$$

## 3. Results

### 3.1. Mapping of the CEI in the Study Area

**Figure 4:**
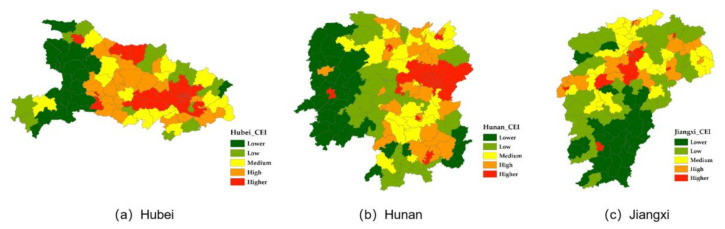
CEI distribution maps of Hubei, Hunan, and Jiangxi in 2018.

Based on the method proposed in 2.3, this study obtained the CEI of each county in Hubei, Hunan and Jiangxi in 2018. According to the statistical distribution of the CEI values, the CEI is divided into five categories by using the quantile classification method: lower (CEI ≤ 0.078), low (0.078 < CEI ≤ 0.112), medium (0.112 < CEI ≤ 0.149), high (0.149 < CEI ≤ 0.219) and higher (CEI > 0.219). The CEI distribution maps of Hubei, Hunan and Jiangxi in 2018 were drawn, and the results are shown in Figure 4.

Generally, in 2018, the CEI values of the counties in the Hubei, Hunan and Jiangxi provinces show the same distribution pattern: the high-value areas are mainly concentrated in the provincial capital and the surrounding urban agglomeration. However, there is an obvious imbalance in the development level among counties. The CEI of the counties in the central part of Hubei Province is significantly higher than that in the western and eastern regions. The regions of Hunan Province with high CEI values are mainly distributed in the north and south sides of Changsha City, and the CEI gradually decreases to the west. The areas of Jiangxi Province with high CEI values is mainly concentrated in the north, with Nanchang as the center, while the regions with low CEI values are mainly distributed in the south. A more detailed spatial analysis will be given in Section 3.3.

To test the validity of the CEI in evaluating economic development, this study randomly selected 32 sample counties from the data set, divided the GDP and TNLI of the sample counties into five grades according to the same classification method, and compared the rank difference between the CEI, GDP and TNLI. Table 5 shows some differences in the rank of sample counties due to more comprehensive evaluation criteria for economic development, such as Zhushan, Badong, Cili and Lianyuan. The CEI rank of most sampled counties is lower than their respective GDP rank, which is mainly because of poor terrain, lack of material capital, inconvenient transportation and poor medical conditions. Therefore, compared with a single-dimension economic parameter, such as GDP, a multidimensional indicator evaluation more comprehensively reflects the economic status of a county. At the same time, we find that, compared with the NPP-VIIRS data, in general, the difference between the LJ1-01 TNLI rank and CEI is more modest, and all the absolute values are no more than 2.

### 3.2. Model Results and Accuracy

**Figure 5:**
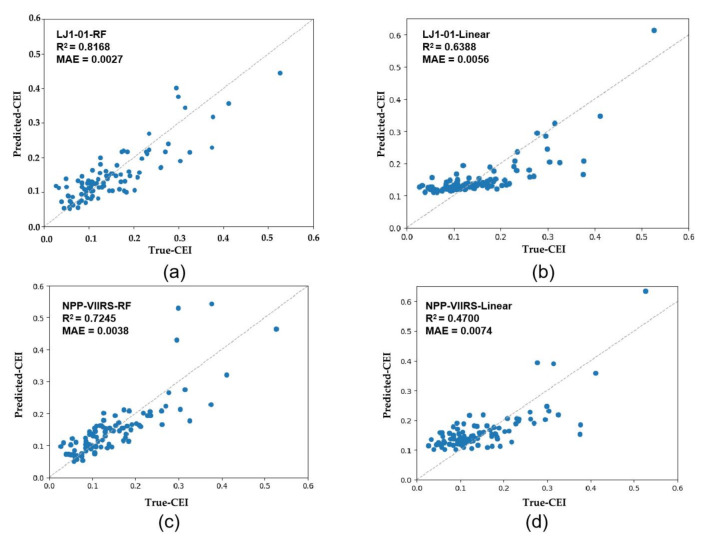
Scatter diagrams between the true CEI and the predicted CEI. (**a**) RF model using LJ1-01 NTL; (**b**) LR model using LJ1-01 NTL; (**c**) RF model using NPP-VIIRS NTL; and (**d**) LR model using NPP-VIIRS NTL.

We randomly selected 70% of the counties in the study area (a total of 325 counties) as the training set, and the remaining 30% of the counties were taken as the test set to train and fit the RF model. After parameter optimization, the final fitting results are obtained. To verify the advantages of RF in estimating multidimensional economic development at the county level, we also used the LR model for comparison. Figure 5 shows the scatter plots between the true CEI derived from the statistics and the predicted CEI estimated through the LR and RF models in the sampled counties. Table 6 compares the model accuracy of the LR and RF. Taking the LJ1-01 NTL data as an example, in the LR model the regression R^2^ is 0.638 and the MAE is 0.0056; whereas, in the RF model, the regression R^2^ is 0.8168 and MAE is 0.0027. It can be seen that the performance of the LR model is relatively poor in comparison with the RF algorithm. The latter is less sensitive to data multicollinearity and is robust in describing complex nonlinear relationships involving multiple variables [50]; thus, better performance can be achieved. At the same time, in the RF model, the R^2^ value for the LJ1-01 data (0.8168) was higher than that for the NPP/VIIRS data (0.7245); the results show that the correlation between the LJ1-01 data and the CEI is obviously higher than the equivalent NPP/VIIRS data. In order to minimize the impact of the error caused by the training set, we performed 10 cross-validations. The results also show that the R^2^ of LJI-01 (0.7920 in the RF model) is higher than that of NPP-VIIRS (0.7054 in the RF model). In other words, the LJ1-01 data have greater modeling potential, which is mainly due to a wider range of radiance values, as well as the higher spatial resolution and capacity of these data to reflect the social activities at night in more detail.

**Figure 6:**
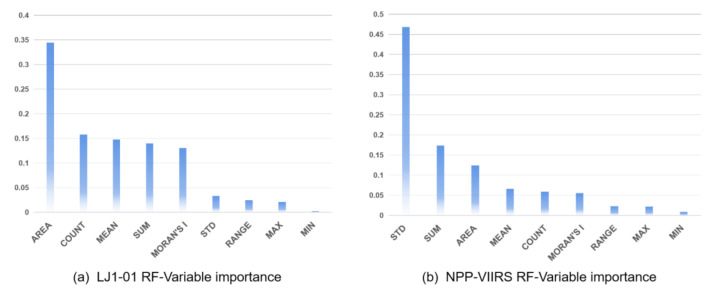
Importance of the nine variables in the RF model: (**a**) LJ1-01 NTL and (**b**) NPP-VIIRS NTL.

The selected variables, ordered by their importance in the RF model, are shown in Table 7 and Figure 6. In the LJ1-01 RF model, the most important variable is AREA, with an importance value of 0.3445. The COUNT, ANLI, TNLI and MORAN’S I are also important, with values all greater than 0.1, whereas in the NPP-VIIRS RF model, the most important variable is the STD, which is 0.4681, followed by the TNLI and AREA, which are the only other two variables with values greater than 0.1. Generally, counties with better economic development will have a larger range of night light area, and in counties with balanced economic development, the distribution of light pixels will be more even. Compared with the NPP-VIIRS data, the LJ1-01 data has more image features with relatively high importance in the RF model. The ranking of variable importance indicates that, compared with the NPP-VIIRS data, the image characteristics of the LJ1-01 data can be better applied to the RF model, which improve the recognition accuracy of the CEI. Whether in the LJI-01 or NPP-VIIRS RF regression model, the importance values of the RANGE, MAX and MIN are relatively small, especially in the LJ1-01 RF model. This is probably because the MIN value in most counties are extremely small, resulting in a small difference between the RANGE and MAX values.

**Figure 7:**
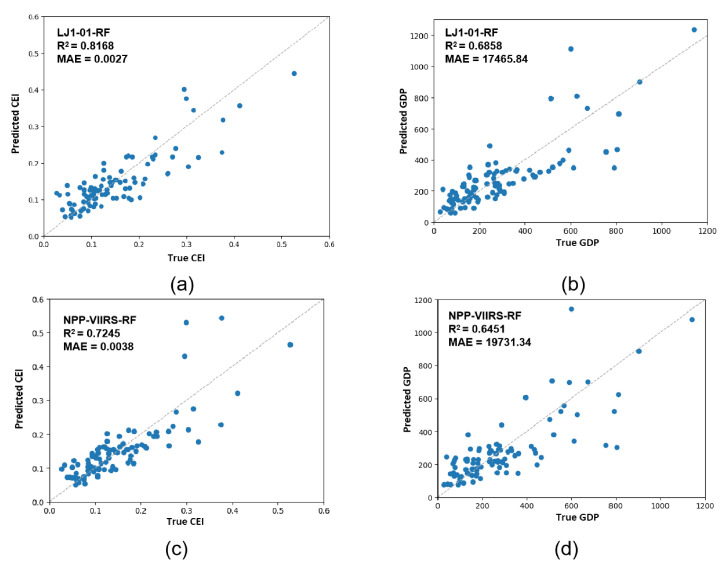
Scatter diagrams of the RF regression model for the CEI and GDP: (**a**) prediction of CEI using LJ1-01 NTL data; (**b**) prediction of GDP using LJ1-01 NTL data; (**c**) prediction of CEI using NPP-VIIRS NTL data; and (**d**) prediction of GDP using NPP-VIIRS NTL data.

To further verify the advantages of applying CEI to estimate county economic development, we also used the RF model to regress the GDP of the sampled counties and found that the R^2^ decreases compared with CEI regression results; the results are shown in Table 8. Figure 7 shows the scatter diagrams of the RF regression model for the CEI and GDP. Taking the LJ1-01 NTL data as an example, the regression R^2^ value of the CEI (0.8168) was significantly higher than that of GDP (0.6858), indicating that, compared with selecting the GDP from the sampled counties, it is more scientific and reliable to use multidimensional indicators to evaluate the economic development of counties. In addition to that, the results are more consistent with the image features of the NTL remote sensing data. Furthermore, it can be found that the regression R^2^ value of the GDP for the LJ1-01 data (0.6858) was higher than that of the NPP/VIIRS data (0.6451). The results also indicate that the LJ1-01 data are to some extent superior to the NPP-VIIRS data in the estimation of socioeconomic parameters. Accordingly, using the LJ1-01 data to detect the county-level economic development is a more feasible and reliable method. The multidimensional county-level economic index evaluated by the NTL data provides a scientific basis for the relevant government departments and can be a good reference for future socioeconomic development strategies.

### 3.3. Spatial Analysis of the CEI

In this study, Moran’s I and LISA were used to analyze the distribution of the CEI in the Hubei, Hunan and Jiangxi provinces. The Moran’s I values for the three provinces are shown in Table 9. The Moran’s I values of the three provinces are all greater than 0 and have been validated by the hypothesis test. The result shows that the CEI distribution of the three provinces presents a positive spatial correlation. Hubei Province has the highest Moran’s I, which is 0.7125. The economic development of counties in this province is extremely unbalanced, while the Moran’s I of Jiangxi Province is relatively small, with a value of 0.4976. The counties within this province present a relatively balanced development state.

**Figure 8:**
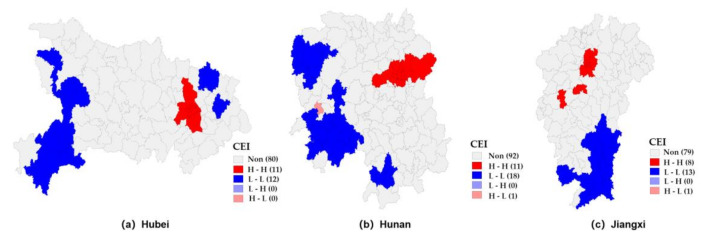
Local indicators of spatial association (LISA) of the CEI in Hubei, Hunan and Jiangxi.

Figure 8 shows the LISA statistics of the CEI values in the Hubei, Hunan and Jiangxi provinces. In general, the high–high clustering regions of the three provinces are concentrated in the metropolitan area surrounding their capital cities. At the same time, each province also presents its own characteristics of CEI distribution. In Hubei Province, the low–low clustering areas are mainly concentrated in the western part of the province (including Enshi, western Yichang and southern Shiyan, where the geographical environment is poor, and thus is unable to benefit its economic development) and in Huanggang City in the eastern part of Wuhan, with limited development and a lack of social materials. In Hunan Province, the low–low clustering areas are mainly concentrated in the southwest of Hunan Province, including the Xiangxi Autonomous Region, southwestern Huaihua, Shaoyang and southern Yongzhou. These areas are also affected by natural conditions and material shortage, and their development is relatively slow. In addition, Hecheng district, the central urban area of Huaihua City, presents a high–low clustering, which indicates that the development focus of Huaihua City is mainly concentrated in the central urban area. Compared with the surrounding counties, the development of this area is obviously unbalanced. In Jiangxi Province, the low–low clustering area is mainly concentrated in Ganzhou City, which is located in the south of the province. In addition to the metropolitan area, with Nanchang as the center, there are two other high–high clustering areas in the east of Yichun City and Xinyu City, which indicates that the economic development of the counties in the north of Jiangxi Province is relatively balanced.

## 4. Discussion

**Figure 9:**
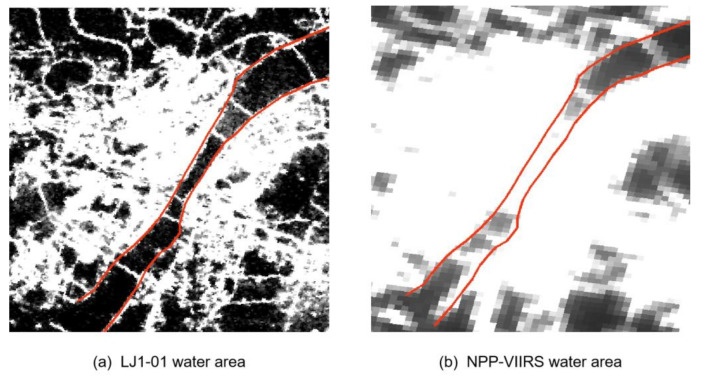
Comparison of the (**a**) LJ1-01 data and (**b**) NPP-VIIRS data in a water area.

In this study, the LJ1-01 and NPP-VIIRS data are important sources of information for nighttime light intensity, and their potential for detecting country-level economic development were investigated. The RF regression model and LR regression model were compared to verify the feasibility and advantages of the machine learning algorithm in the application of nighttime light data. The comparison of the two types of NTL data shows that the LJ1-01 data offered higher accuracy and more potential than the NPP-VIIRS data in terms of modeling multidimensional socioeconomic indicators (Table 6 and Table 8). This is mainly due to the higher spatial resolution provided by the LJ1-01 data, which better captures the finer spatial details of artificial lighting than the NPP-VIIRS data, leading to more detailed characteristics of the social activities at night. The improved spatial resolution enables the LJ1-01 data to exhibit a more consistent spatial pattern of county economic development with the reference data. Furthermore, compared with the NPP-VIIRS data, the LJ1-01 images have fewer blooming phenomena. Previous studies have shown that the NPP-VIIRS data improved the spatial resolution and reduced blooming compared with DMSP-OLS composites [51]. Similarly, we found that LJ1-01 had the same advantage over the NPP-VIIRS composites. According to the research of Elvidge et al. [52], the blooming effect can lead to overestimation of urban economic parameters. Thus, the LJ1-01 nighttime light imagery has a stronger capacity to detect economic development. This conclusion can also be verified by the importance ranking of the variables obtained in this study (Figure 6). Compared with the NPP-VIIRS images, the RF regression model can do better to identify the characteristics of the light area and the count of light pixels in the LJ1-01 images; additionally, the importance of the minimum value of the county light pixels in the LJ1-01 images is minimal. Figure 9 takes the water body of the Yangtze River in Wuhan, which is a typical region with few human activities and low NTL intensity, as an example to compare the light radiation values of NPP-VIIRS and LJ1-01. The radiance values of NPP-VIIRS in the Yangtze River all exceed 0, whereas those of LJ1-01 are 0, especially in the center of the Yangtze River. This discovery provides a reference for future research.

In addition, this paper analyzed the county economic patterns of the Hubei, Hunan and Jiangxi provinces. The same regression method was implemented separately for the three provinces. The results are shown in Table 10. Hubei Province has the highest accuracy (R^2^ = 0.8610 using LJ1-01 NTL to regress the CEI of Hubei; R^2^ = 0.8388 using NPP-VIIRS NTL to regress the CEI of Hubei). Due to the large gap in economic development among the counties, Hubei Province has a wide range of CEI values (from 0.0246 to 0.7513), which is more suitable for detecting the relationship between the CEI and nighttime light imagery. Additionally, the overall level of county economic development in Hubei Province is relatively higher; thus, the correlation between the CEI and the NTL data is closer.

In summary, the LJ1-01 data can be used as an effective tool to establish socioeconomic indicator models for evaluating a county’s economy. Nevertheless, it is also acknowledged that there are still some limitations for the application of LJ1-01 NTL data. First, due to the spectral bandpass of LJ1-01, the blue sensitivity of LJ1-01 decreases at 460 nm, which will miss some fraction of spectral power in the 450 nm feature of blue-pump white LEDs. Therefore, it is difficult for LJ1-01 to see some short wavelength contribution, such as the blue light from white LED emissions, which may affect the experimental results. Secondly, the images prior to June 2018 are vacant, which limits the application of these data in the time series analyses to a certain extent, especially for analyses of historical information related to urban dynamics. Thirdly, the LJ1-01 data contain slight geo-referencing errors, which constrain the direct and accurate application of these data in spatial analysis. The preprocessing of high-precision geometric correction is essential for LJ1-01 NTL to reduce geometric positioning errors. Although the Chinese 2018 synthetic LJ1-01 NTL data applied in this paper improved the positioning accuracy, the data only comprise a single image and cannot be analyzed in a long time series. Finally, the quality of some LJ1-01 images is also affected by clouds and moonlight, which will result in errors in remote sensing applications.

## 5. Conclusions

LJ1-01 is a new generation of NTL satellites with a finer spatial resolution and freely available data. Its NTL imagery has great potential for studying socioeconomic issues. In this study, the nighttime light data obtained from the LJ1-01 and NPP-VIIRS satellites were used as data sources to detect the economic development of counties in the Hubei, Hunan, and Jiangxi provinces. By selecting multidimensional economic indicators, the county-level economic index (CEI) is calculated using the Entropy-AHP method. Then, the relationship between the CEI and NTL imagery is explored by using LR and RF regression models. The results show that compared with the traditional linear regression algorithm, the RF algorithm can significantly improve the accuracy of the model. In addition, compared with the regression results using the NPP-VIIRS data, results estimated by the LJ1-01 data have higher determinate coefficients, indicating the improved potential of the LJ1-01 data in estimating socioeconomic parameters. This study also detected the spatial pattern of the CEI in the Hubei, Hunan and Jiangxi provinces and found that Hubei Province has the highest and most unbalanced development level among the three provinces, which further proves the feasibility and efficiency of nighttime light imagery in county-level economic development evaluation. In conclusion, this study confirmed that, combined with machine learning algorithms, the LJ1-01 data present a great potential for detecting county-level economic development.

In future research, the integration of LJ1-01 nighttime imagery and multisource data, such as other satellite remote sensing images or fine-scale socioeconomic attributes, may provide more possibilities for accurate identification of human activities. In addition to the RF regression model, more machine algorithms, such as SVM and the Gaussian process, can be applied for future studies using the LJ1-01 data. In addition, since there are still some challenging issues in the LJ1-01 imagery, such as the limitation of the spectral bandpass, geometric positioning errors and the influence of moonlight and clouds, further research is required to improve the quality of the data for wide application.

## Figures and Tables

**Table 1:** Specifications of the three types of nighttime light (NTL) data.

	DMSP-OLS	NPP-VIIRS	LJ1-01
**Time Span**	1992–2013	November 2011–present	June 2018–March 2019
**Swath**	~3000 km	~3000 km	~250 km
**Spectrum**	0.5–0.9 μm	0.5–0.9 μm	0.46–0.98 μm
**Quantization**	6 bits	14 bits	14 bits
**Spatial Resolution**	2700 m	750 m	130 m
**Calibration**	N/A	On-board Calibration	On-board Calibration
**Saturation**	Saturated	Not Saturated	Not Saturated

**Table 2:** Evaluation indicators used to calculate the County-level Economic Index (CEI).

Dimension	Indicator	Attribute	Weight	Interpretation of the Indicator
**Economic Conditions**	County GDP	+	0.2494	Reflects the overall state of economic development in the county
	Proportion of secondary industry	+	0.0062	Percentage of the secondary industry in GDP
	Proportion of tertiary industries	+	0.0075	Percentage of the tertiary industry in GDP
	County fiscal revenue	+	0.2108	Reflects the financial level of the government in the county
	County total retail sales of consumer goods	+	0.1097	Reflects the consumption level of residents in the county
**People’s Livelihood**	Urban per capita disposable income	+	0.0197	Reflects the income and living standards of urban residents in the county
	Rural per capita disposable income	+	0.0418	Reflects the income and living standards of rural residents in the county
	Urban per capita living space	+	0.0042	Reflects the housing conditions of urban residents in the county
	Rural per capita living space	+	0.0022	Reflects the housing conditions of rural residents in the county
**Social Development**	Urbanization rate	+	0.1297	Reflects urbanization level in the county
	Urban unemployment rate	-	0.0095	Reflects the employment situation of the resident in the county
	Population density	+	0.1150	Reflects the density of population in the county
**Public Resources**	Number of students per 10,000 people in general primary and secondary schools	+	0.0080	Reflects educational conditions in the county
	Number of beds per 10,000 people in health institutions	+	0.0212	Reflects medical conditions in the county
	Road network density	+	0.0065	The ratio of the total length of roads at all levels and area of the county, reflecting traffic conditions in the county
**Natural Vulnerability**	Potential productivity of farmland	+	0.0092	Reflects the potential of agriculture production in the county
	Average slope	−	0.0144	Reflects terrain conditions in the county
	Area with slope greater than 15°	−	0.0342	Reflects terrain conditions in the county
	Average precipitation	−	0.0007	Reflects climate conditions in the county; high precipitation is detrimental to agricultural production in the study area
	Average temperature	+	0.0001	Reflects climate conditions in the county; low temperatures are detrimental to agricultural production in the study area

**Table 3:** Relationship between the randomness index (RI) and the judgment matrix order.

**N**	2	3	4	5	6	7	8	9	10
**RI**	0	0.58	0.9	1.12	1.24	1.32	1.41	1.45	1.49

**Table 4:** Feature indicators of the nighttime light data.

Dimension	Feature Description	Feature Indicator	Calculation
**Central tendency**	Average nighttime light index (ANLI) in the county	MEAN	$\sum_{i=1}^{N}x_{i}/N$
	Total nighttime light index (TNLI) in the county	SUM	$\sum_{i=1}^{N}x_{i}$
**Dispersion degree**	Range of light value in the county	RANGE	MAX–MIN
	Standard deviation of light value in the county	STD	$\sqrt{\frac{\sum_{i=1}^{N}{\left(x_{i}-\bar{x}\right)}^{2}}{N-1}}$
**Spatial characteristics**	Maximum light value in the county	MAX	–
	Minimum light value in the county	MIN	-
	Number of bright light pixels (*DN* > 0) in the county	COUNT	-
	Relative light area in the county	AREA	$S_{\mathrm{light}}/S_{\mathrm{total}}$
	Local Moran’s index of light value in the county	MORAN’S I	$\frac{\sum_{j=1,j\neq i}^{n}w_{ij}(y_{i}-\bar{y})(y_{j}-\bar{y})}{\sum_{i=1}^{n}{\left(y_{i}-y\right)}^{2}/n}$

**Table 5:** Comparison of class ranks between the CEI, GDP and TNLI.

County	CEI Rank	GDP Rank	TNLI-VIIRS Rank	TNLI-LJ1 Rank	Rank Difference CEI vs. GDP	Rank Difference CEI vs. TNLI-VIIRS	Rank Difference CEI vs. TNLI-LJ1
Wuchang	5	5	5	5	0	0	0
Yangxin	4	3	5	3	1	−1	1
Yunxin	1	1	3	1	0	−2	0
Zhushan	1	2	3	2	−1	−2	−1
Zigui	1	2	2	1	−1	−1	0
Zhongxiang	4	5	5	4	−1	−1	0
Hanchuan	5	5	5	5	0	0	0
Gongan	4	4	4	3	0	0	1
Wuxue	4	4	4	4	0	0	0
Lichuan	2	2	5	4	0	−3	−2
Badong	1	2	4	2	−1	−3	−1
Laifeng	1	1	2	1	0	−1	0
Liuyang	5	5	5	5	0	0	0
Chaling	2	3	2	2	−1	0	0
Xiangtan	4	5	4	4	−1	0	0
Changning	3	4	3	3	−1	0	0
Xinshao	2	2	3	2	0	−1	0
Dongkou	2	3	2	1	−1	0	1
Yueyang	3	4	4	3	−1	−1	0
Linxiang	3	4	3	3	−1	0	0
Wulin	5	5	5	5	0	0	0
Yongding	3	3	5	3	0	−2	0
Cili	2	3	4	1	−1	−2	1
Taojiang	3	4	3	3	−1	0	0
Linwu	2	2	2	2	0	0	0
Rucheng	1	1	2	2	0	−1	−1
Shuangpai	1	1	1	1	0	0	0
Lianyuan	2	4	3	3	−2	−1	−1
Changjiang	4	4	3	4	0	1	0
Leping	4	4	3	4	0	1	0
Yushui	5	5	5	5	0	0	0
Jingan	1	1	1	1	0	0	0
Yushan	3	3	2	3	0	1	0

In this table, 1 represents a lower class, 2 represents a low class, 3 represents a medium class, 4 represents a high class and 5 represents a higher class.

**Table 6:** Comparison of accuracy between the LR and RF models.

Methods	LR Model		RF Algorithm	
**Data**	**LJ1-01**	**NPP-VIIRS**	**LJ1-01**	**NPP-VIIRS**
**R^2^**	0.6380	0.4700	0.8168	0.7245
**MAE**	0.0056	0.074	0.0027	0.0380
**10-times cross-validated R^2^**	0.6215	0.4533	0.7920	0.7054

**Table 7:** Importance of each variable for LJ1-01 and NPP-VIIRS in the RF model.

ID	Variable	LJ1-01	NPP-VIIRS
1	AREA	0.3445	0.1241
2	COUNT	0.1581	0.0593
3	MEAN	0.1474	0.0664
4	SUM	0.1399	0.1732
5	MORAN’S I	0.1301	0.0556
6	STD	0.0331	0.4681
7	RANGE	0.0246	0.0232
8	MAX	0.0211	0.0216
9	MIN	0.0023	0.0088

**Table 8:** Comparison of the accuracy for the RF model to regress CEI and GDP.

Index	CEI		GDP	
**Data**	**LJ1-01**	**NPP-VIIRS**	**LJ1-01**	**NPP-VIIRS**
**R^2^**	0.8168	0.7245	0.6858	0.6451
**MAE**	0.0027	0.038	17,465.84	19,731.34

**Table 9:** Moran’s I values for the Hubei, Hunan and Jiangxi provinces.

Province	Moran’s I	Z Score	*p*-Value
Hubei	0.7125	11.710	<0.001
Hunan	0.6641	11.967	<0.001
Jiangxi	0.4976	8.053	<0.001

**Table 10:** The accuracy of the RF regression (R^2^) in the Hubei, Hunan and Jiangxi provinces.

Province	LJ1-01	NPP-VIIRS	Range of CEI
Hubei	0.8610	0.8388	0.0246–0.7513
Hunan	0.7832	0.7286	0.0393–0.6016
Jiangxi	0.7647	0.7082	0.0297–0.3547

## Appendix A

The following notations are used in this paper and is defined where first used in the text. For the explanation of the notations, refer to Table A1. The data applied in this paper and the related instructions are shown in Table A2.

**Table A1:** Notations used in this paper.

Notation	Explanation
*DN*	The light radiation value
i,j	The i-th or j-th index
A	The judgment matrix of AHP
a_ij_	Scalar, representing matrix elements
W	The weight vector
CI	The consistency verification index in AHP
CR	The consistency ratio in AHP
RI	The randomness index in AHP
P_ij_	The dimensionless value
*e_i_*	The entropy value
*g*_i_	The coefficient of variation
X_i_	Scalar, representing the corresponding index value
x_i_	Scalar, representing the corresponding value at spatial point i
$\bar{x}$	Scalar, representing the corresponding average value
R^2^	The determinate coefficients
MAE	The mean absolute error
I	The value of Moran’s I
I_i_	The value of LISA statistic

**Table A2:** Data sources used in this article and related instructions.

Data Type	Data Name	Data Source	Instruction
Nighttime Light Data	NPP-VIIRS NTL data	The Observation Group, Payne Institute for Public Policy (https://eogdata.mines.edu/download_dnb_composites.html)	48 phases of monthly composite data from 2015 to 2018 and 2 phases of annual NTL data from 2015 to 2016. The data needs to eliminate background noise.
	LJ1-01 NTL data	High-Resolution Earth Observation System of the Hubei Data and Application Center (http://www.hbeos.org.cn/)	The Chinese 2018 synthetic LJ1-01 NTL data have been adjusted based on ground control points. The data needs absolute radiation correction.
Socioeconomic Statistical Data	The Statistical Yearbook of 2019	The provincial statistical bureaus (http://tjj.hubei.gov.cn/, http://tjj.hunan.gov.cn/, http://tjj.jiangxi.gov.cn/)	15 socioeconomic parameters such as county GDP, County fiscal revenue, Per capita disposable income, Urbanization rates, etc., were selected from the four perspectives of economic conditions, people’s livelihood, social development and public resources.
	The 2018 National Economic And Social Development Statistical Bulletins published by each county		
Basic Geographic Information Data	The administrative boundary	The Resource And Environment Data Cloud Platform of the Chinese Academy of Sciences (http://www.resdc.cn/Default.aspx)	All the basic geographic data were re-projected to the Albers equal-area projection coordinate system and resampled at 1000 m × 1000 m.
	DEM		
	Farmland production potential data		
	Monthly average precipitation data	The National Earth System Science Data Center, National Science and Technology Infrastructure of China (http://www.geodata.cn)	
	Monthly average temperature data		
